# African Swine Fever: Lessons to Learn From Past Eradication Experiences. A Systematic Review

**DOI:** 10.3389/fvets.2020.00296

**Published:** 2020-06-09

**Authors:** Maria Luisa Danzetta, Maria Luisa Marenzoni, Simona Iannetti, Paolo Tizzani, Paolo Calistri, Francesco Feliziani

**Affiliations:** ^1^National Reference Centre for Veterinary Epidemiology and Risk Analysis (COVEPI), Istituto Zooprofilattico Sperimentale dell'Abruzzo e del Molise, G. Caporale, Teramo, Italy; ^2^Department of Veterinary Medicine, University of Perugia, Perugia, Italy; ^3^World Animal Health Information and Analysis Department (WAHIAD), World Organisation for Animal Health, OIE, Paris, France; ^4^National Reference Laboratory for Swine Fevers, Istituto Zooprofilattico Sperimentale dell'Umbria e delle Marche “Togo Rosati”, Perugia, Italy

**Keywords:** African swine fever, data sharing, emergency preparedness, eradication, risk factors, surveillance, systematic review

## Abstract

Prevention, early detection, prompt reaction, and communication play a crucial role in African swine fever (ASF) control. Appropriate surveillance capable of early detection of the disease in both domestic and wild animals, and the implementation of consolidated contingency plans, are currently considered the best means of controlling this disease. The purpose of this study was to understand the lessons to be learned through the global disease eradication history. To establish which strategies were successful for prevention, control, and eradication of ASF, and which errors should not be repeated, we conducted a systematic review. A query was defined to search for surveillance and control strategies applied by countries worldwide for ASF eradication in the past. Inclusion and exclusion criteria were defined. Decisions on study eligibility and data extraction were performed by two independent reviewers and the differences were resolved by consensus or by a third reviewer. From 1,980 papers, 23 were selected and included in the qualitative analysis. Reports from Belgium, Brazil, Cuba, the Dominican Republic and Haiti, France, mainland Italy, Malta, Portugal, and Spain were included. Despite the economic resources allocated and the efforts made, eradication was possible in only eight countries, between the 50s and 90s in the twentieth century, in different epidemiological and cultural contexts, in some instances within <1 year, and in others in about 40 years. Classical surveillance strategies, such as active and passive surveillance, both at farm and slaughterhouse levels, targeted surveillance, together with conventional biosafety and sanitary measures, led to eradication even in countries in which the tick's epidemiological role was demonstrated. Historical surveillance data analysis indicated that eradication was possible even when technological tools either were not available or were used less than they are currently. This emphasizes that data on surveillance and on animal population are crucial for planning effective surveillance, and targeting proper control and intervention strategies. This paper demonstrates that some strategies applied in the past were effective; these could be implemented and improved to confront the current epidemiological wave. This offers encouragement for the efforts made particularly in Europe during the recent epidemics.

## Introduction

The causative agent of African swine fever is a unique member of the *Asfarviridae* family, the *Asfivirus* (ASFV) (1); a genetically complex double-stranded DNA virus that contains a series of genes related to virulence, immune evasion, and cell process modulation (2). Twenty-three genotypes have been described based on the partial sequences of the p72 gene (3, 4). All 23 genotypes are present in Africa, whereas only genotypes I and II have been found outside of that continent. ASF is a notifiable disease in the European Union (EU) and should be reported to the World Organization for Animal Health (OIE). Due to the related impact on international trade in live animals and swine products and the socio-economic consequences on individuals' livelihoods, the disease remains a major concern for infected and non-infected countries, as there is no effective treatment and effective vaccines are not still available (5, 6).

The virus can affect species of the Suidae family (both wild and domestic) of all breeds and ages. Virulent ASFV strains cause peracute or acute hemorrhagic fever in infected animals, with up to 100% mortality (7). Generally, clinical disease can manifest in multiple ways, ranging from death, with no signs (peracute, mortality nearing 100%), to an asymptomatic infection; however, most isolates of ASFV cause acute hemorrhagic fever in domestic pigs and result in mortality nearing 100% (8, 9). European wild boar (*Sus scrofa*) is highly susceptible to the disease and shows similar clinical signs and lethality as domestic pigs (*Sus scrofa domesticus*). In contrast, infected wild African suids usually have occult infections and develop subclinical and asymptomatic long-term persistent infections, acting as ASFV reservoirs in Africa.

ASFV is primarily transmitted via direct and indirect contact between animals, through infected swine and their products, and via contaminated fomites or uncooked meat from infected animals. Its ability to persist for a long time in the environment or in infected biological samples makes eradication difficult once the disease has become established. Additionally, some arthropods that may have acquired ASFV during preceding years (up to 5 years) can transmit the virus (10). Soft ticks of the *Ornithodoros* spp. can be an effective reservoir of infection (8, 11), with documented trans-stadial, trans-ovarial, and sexual transmission (12). However, these tick species have not been shown to be involved in transmission of ASFV in Eastern Europe, Russia, or the trans-Caucasus region (13), whereas potential sources of infection in Europe are represented by infectious domestic pigs (*Sus scrofa domesticus*) and wild boar (*Sus scrofa*), contaminated carcasses, food waste, and vehicles or equipment. Furthermore, in Sardinia (Italy), where the disease has been persisting for more than 35 years, recent studies have reaffirmed the absence a role of *O. erraticus* ticks in the ASF cycle, despite strong climatic and ecological similarities with the Iberian Peninsula, where this tick was involved in ASFV transmission and the persistence of ASF (14, 15).

In addition to the presence of carrier animals (16), there are several other mechanisms that can lead to long-term circulation of ASFV in pig or wild boar populations. The most important are human-induced factors, such as illegal movement of infected pork and swill feeding (16–23), as well as free-range pig management systems as it was observed in some regions of Russia (18, 21).

ASF was confined to Africa until the end of the 1950's, when Genotype I ASFV strains first appeared in Portugal, in 1957, probably via a single-source introduction from Angola (24). This epidemic wave involved different countries in Europe and then also in some Central and South American countries. After the virus introduction into the Russian Federation in 2007 (20), in order to mitigate the risk of ASFV spread toward the EU, the EU Member States bordering the Russian Federation implemented specific protection measures. Despite this, in 2014 ASF entered Estonia, Latvia, Lithuania, and Poland, where the disease has become endemic in the wild boar population (25), whereas the sporadic outbreaks occurring in domestic pigs have been efficiently controlled, thus preventing extensive secondary spread (26). However, in 2016 ASFV spread into Moldova and in 2017 it was reported for the first time in Czech Republic, Romania (27), Bulgaria, and Hungary (28). In September 2018 the virus made a big leap, infecting hundreds of wild boars in Southern Belgium, in a well-limited and confined area of the Walloon region (28). There were also large outbreaks in Asia, starting in China, where a wide part of the territory has been infected since August 2018. In July 2019 the disease was notified for the first time in Slovakia and a month later, in August 2019 (28), it appeared for the first time in Serbia (28).

Currently, the disease is present in more than 20 sub-Saharan African countries (29), in some islands of the Indian Ocean (Madagascar and Mauritius), and from 2007 in some Eastern, Central European countries and in eight countries belonging to the European Union (Lithuania, Polonia, Latvia, Estonia, Romania, Belgium, Slovakia, the island of Sardinia in Italy). In this alarming context, the positive resolution of an outbreak that occurred in a wild boar population resident in a restricted area of the Czech Republic should be considered (30). Nevertheless, there is great concern about the spread of ASFV infection in Asia: after the first occurrences of the disease in China, a number of bordering countries notified many outbreaks and the epidemiological situation appears far from being effectively controlled (31).

The sole European territory where ASF Genotype I (vp72) has been present for a long time is the Italian island of Sardinia (32). The same genotype has been present in Spain and Portugal from 1960 to 1995, and caused outbreaks in some other European countries [France (1964, 1967, and 1977), Belgium (1985), Italy (1967, 1980) Malta (1978), and the Netherlands (1986)] (33). This genotype was also responsible for several outbreaks in the Caribbean and South America (from 1971 until 1981) (34). Since 1995, all affected European and south American countries had successfully eradicated the disease (32), with only Sardinia being the exception. On the other hand, all ASFV isolates circulating in Azerbaijan, Armenia, the Russian Federation, in other Eastern and Central European countries since 2007, are all clustered within Genotype II (29).

ASF epidemiology is thus very complex, determining different epidemiological patterns of infection when considering Africa or Europe. From an epidemiological point of view, three independent epidemiologic cycles (sylvatic, tick–pig, and domestic) have been described (35) until recently in literature. After the ASF epizootic occurred in Central and Eastern EU Member States, the researchers could consider a fourth cycle in addition to the three already recognized: the “wild boar–habitat cycle” (36). This cycle focuses on the wild boar population and its habitat as a virus reservoir (37). Different epidemiological scenarios can be outlined according to the geographical area, the species involved, the transmission route, and the risk factors identified for ASF persistence and spread (Table 1).

**Table 1:** ^a^Epidemiological scenarios, by geographical area.

**Geographical area**	**Species involved**	**Route of transmission**	**Risk factors for persistence or spread**	**Other areas with an overlapping scenario**
Eastern and Southern African countries (currently)	Wild suids (asymptomatic *Phacochoerus* and *Potamochoerus* spp.), Soft ticks (*O. moubata* as reservoir) Domestic pigs (34)	Sylvatic warthog–tick cycle and/or domestic-tick or domestic pig cycle (38). Transmission to domestic pigs through the **bite of infected ticks** and the ingestion of tissues from acute-infected warthogs. Movement of infected pigs and products (38).	**Low biosecurity in pig farms**, marketing of infected pigs and products, cultural constrains (38), human behavior (8). Relevant role of soft tick and wild pigs in the maintaining of the disease.	N.A.
West African countries (currently)	Domestic pigs. Ticks suspected not to be involved A sylvatic cycle has never been demonstrating (34, 39).	**Direct contact** between domestic pigs (infected-not infected) **Indirect contact** between not infected pigs and infected pork products	Socioeconomic factors: **lack of compensation** to farmers (underreporting); **lack of veterinary services, low biosecurity farms** with home slaughter with indiscriminate disposal of pig viscera**, swill feeding, illegal selling** of infected pigs and pork products, **cultural practices** (39).	The same as in some areas of the Caucasus and the Russian federation
Russian Federation and trans-Caucasian countries (currently)	Domestic pigs and wild suids (*Sus scrofa*)	Movement of infected/carrier animals (**direct contact between wild boars and domestic pigs**) Transmission **within wild boar population**. Movement of **infected products**.	**Lack of compensation** for slaughtered animals; lack of resources for adequate control measures; **lack of traceability; delays in identification** of new cases; **non-compliance** with **movement bans**; farms with **poor biosecurity**.	N.A.
Sardinia (currently)	Domestic pigs, and wild suids (*Sus scrofa*) No ticks found	Movement of infected/carrier animals (**direct contact between domestic pigs and wild boars/non-registered domestic pigs)**.	Arduous natural **habitats** (hard access). Traditional breeding practices (free ranging pigs or “brado” illegally maintained in demanial areas) (40).	N.A.
Baltic Republics^b^	Mainly wild suids (*Sus scrofa*) Domestic pigs	Uncontrolled movement of infected pigs, pigswill with ASFV. Spread through the continuous wild boar population habitat. **Direct/indirect contact between domestic pigs and wild boars** (41).	Contamination of wooded areas where **infected carcasses of dead wild boars lied for several months**. Association between the number of settlements, the human population size as well as the number of domestic pigs and pig farms, roads, forest cover percentage, and the presence of ASF in wild boar (26). **Long jumps** spread in wild boars likely **by human activity** (38) Lithuania: lack of biosecurity in the non-commercial pig farms (41). Estonia: contaminated fomites, vehicles, or clothing of farm workers (41).	N.A.
Eastern Europe^c^	Mainly domestic pigs Wild suids (*Sus scrofa*) No ticks found		**Small/backyard pig farms** (21, 38). Involvement of humans in the disease spread in Poland, Bulgaria (41).	N.A.

a *The table was created by the use of information (modified and updated) provided by Sánchez-Vizcaíno et al. (7)*.

b ***Baltic Republics:** Latvia, Lithuania, Estonia*.

c ***Eastern Europe:** Belarus, Bulgaria, Hungary, Moldova, Ukraine, Slovakia, and Poland (belonging to Central Europe)*.

All the current applicable control and eradication measures at local level are based on classical disease control methods, including surveillance (active/passive, targeted to domestic/wild species), epidemiological investigation, pig tracking, and stamping out the virus in infected holdings. All these measures are combined with strict quarantine and biosecurity measures in domestic pig holdings and by the control of animal movement. Early disease detection both in wild and in domestic pigs is considered to be crucial to maintaining an ASF-free health status and is the most complex facet of effective disease surveillance.

The main purpose of this review was to study the ASF eradication history, in order to highlight effective strategies applied for ASF surveillance, control, and eradication in countries that succeeded in stamping out the disease, and to identify what are possible gaps currently hampering ASF control and eradication.

## Materials and Methods

### Literature Sources and Search Strategy

The literature search was performed by querying PubMed, Web of Science, and Scopus databases to retrieve all papers (“primary sources of information”) that could be included in the process of identification, screening, and final eligibility. Additional papers were found by manual searching or by screening the primary sources of information. The platforms were queried by means of Boolean operators, including the search terms (African swine fever OR ASF virus) AND (epidemiology OR spatial pattern^*^ OR temporal pattern^*^ OR trend^*^ OR “control measures” OR control^*^ OR eradication^*^).

The query was searched in “all fields” to allow the retrieval of articles in which the terms appeared in the titles, abstracts, or keywords. Moreover, a filter on the geographical area/territories/countries was applied to exclude the African continent, and the time frame selected was from 1st January 1960 to 31st October 2019.

**Figure 1:**
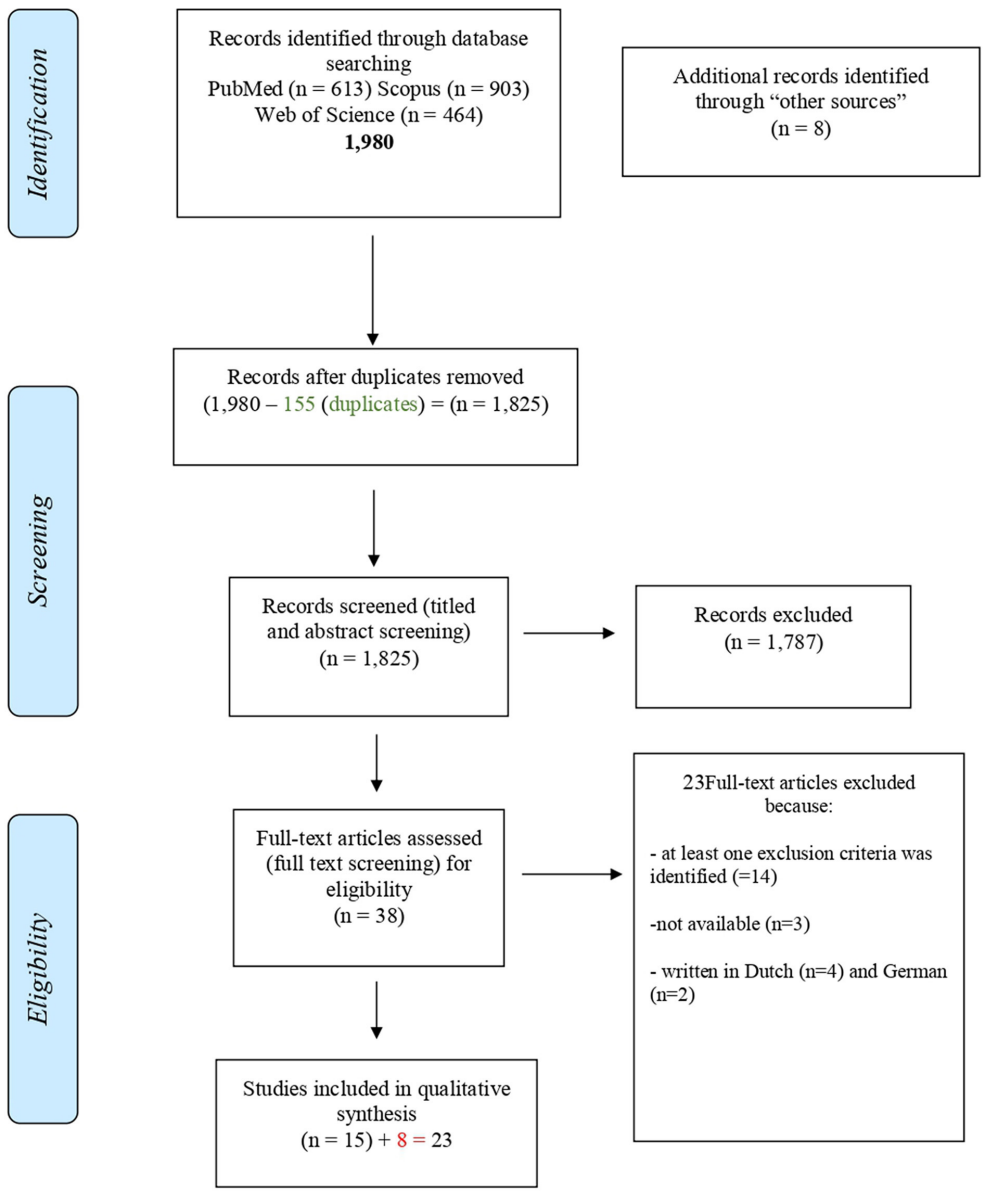
PRISMA flow chart schematizing the process for paper identification, screening, and eligibility determination (42).

Inclusion and exclusion criteria were defined on the systematic review aims and objectives. A PRISMA flow chart was used to map out the number of records identified, included, and excluded, and the reasons for exclusions in each step of the screening process were described (Figure 1).

Studies were initially selected through a search of the titles and abstracts (first screening), and then by reading the full texts (second screening). Decisions on study eligibility and data extraction were performed by two independent reviewers, using electronic forms, and differences between the reviewers were resolved by discussion to consensus or by consulting a third reviewer. References were managed in RefWorks.

During the reading of the title and abstract, the papers were judged ineligible for further screening in full text if they were clearly referring to diseases other than ASF, or at least one of the exclusion criteria was clearly met, in which case, the paper was eliminated.

Each paper identified and included in the previous step was considered eligible for data analysis during the second screening step if fulfilled at least one inclusion criterion.

Information was collected on the dates of first occurrence and eradication of ASF, the type of intervention strategies implemented and the surveillance strategies applied for each country, the risk factors contributing to ASF appearance and its persistence before the eradication goal was met.

### Secondary Sources of Information

Additional information was considered if new papers (in addition to the primary sources) were retrieved by reading the primary sources of information or by manual searches. Secondary information sources were considered in the analysis to ensure inclusion of all available past literature by including additional papers not directly found by the primary searches. The additional papers found as supplementary source of information were used if they met the eligibility criteria or if they complemented some information already achieved through the primary source of information. They were included as “other sources” within the PRISMA Flow Diagram in the identification section (Figure 1).

### Inclusion and Exclusion Criteria

Papers were included in the screening process if they dealt with control and surveillance strategies applied by specific countries to eradicate ASF; if they described control-eradication measures put in place to face and then to eradicate ASF; if they were epidemiological studies and/or studies aimed at designing surveillance and control strategies; studies on transmission dynamics aimed at designing and improving control measures and surveillance in countries where the disease was eradicated; studies aimed at defining or suggesting surveillance and control strategies in countries where the disease was eradicated; or were reviews on surveillance and control strategies applied by countries that achieved eradication. All the articles dealing with ASF surveillance and control measures in countries where the disease was eradicated, they were included. Articles written in English, French, Spanish, and Italian were included.

Studies were excluded if they were performed in the African continent, were outbreak notifications, prevalence studies, description of clinical disease, were studies on pathogenicity and diagnosis, experimental infections in animals and ticks, described research on vaccine development, genome sequencing, if not relevant to the surveillance purposes of ASF; were reviews, if not dealing with surveillance/control and eradication measures, or if dealt with, these were not focused on ASF or were not described in detail; were qualitative and quantitative risk assessments, if these did not target ASF eradication, or papers for which full text was not available. All the articles dealing with ASF surveillance and control measures in countries where the disease was not eradicated, they were excluded. All the articles written in languages other than English, French, Spanish, and Italian were excluded.

### Term Definition

Surveillance strategies were defined as all strategies aimed at collecting, collating, and analyzing information related to animal health and the timely dissemination of information so that action could be taken, according to the Terrestrial Animal Health Code's definition (43). For the purpose of this study, all these strategies aimed at detecting ASF outbreaks and demonstrating freedom from ASFV circulation were considered under the “surveillance strategies” umbrella.

Intervention strategies were defined as all the actions put in place to prevent or reduce the likelihood of ASFV introduction and spread (within and between farms, after having identified the index case) and those aimed at eliminating (eradicating) the sources of virus, according to the definition provided by Guinat et al. (44). They also included biosecurity measures (segregation, cleaning, and disinfection).

## Results

A total of 1,980 papers were found in the databases searched as primary sources of information. After the duplicates were removed (*n* = 155), 1,825 papers were selected for the first screening of titles and abstracts. Of these, 1,787 were excluded by the following criteria: dealing with diseases other than ASF (*n* = 729), type of publication (studies on ASF pathogenicity and diagnosis, experimental infections in animals and ticks with ASFV, communications on clinical findings, *n* = 1,058).

Thirty-eight studies were selected for the second screening by reading of full texts. After the application of the eligibility criteria, 23 papers were excluded because:

- Fourteen met the exclusions criteria: studies on pathogenicity and diagnosis, *n* = 9; papers not dealing with surveillance and control strategies applied for eradication, *n* = 5,
- Three full texts were not available
- Six were written in languages other than the included languages: Dutch (*n* = 4), German (*n* = 2).

Fifteen studies were selected for eligibility from primary sources of information and eight studies from secondary sources of information were added. Finally, 23 papers dealing with the surveillance and control strategies applied for eradication of ASF by specific countries in the past were considered as “eligible” (Figure 1; Table 2) (44).

**Table 2:** Papers included in the review process (PS, primary sources; SS, secondary sources).

**Country**	**Title**	**Platform searched**	**Source type**	**References**
Belgium	“An epizootic of African swine fever in Belgium and its eradication”	PubMed	PS (article)	(45)
Brazil	“The eradication of African swine fever in Brazil, 1978–1984” (article in Spanish)	PubMed; Web of Science	PS (article)	(46)
	“Eradication of African swine fever from Brazil”	By analyzing PS	SS (article)	(47)
	“Epizootiology, laboratory and virulence analyses during the emergency phase of the African swine fever eradication program in Brazil in 1978: a historic account”	PubMed	PS (article)	(48)
	“An analysis of the 1978 African swine fever outbreak in Brazil and its eradication”	PubMed	PS (article)	(49)
Cuba	“Preliminary Report on the African Swine Fever Epizootic in Cuba Methods of diagnosis and control”	PubMed	PS Communication by the Director General—National Institute of Veterinary Medicine	(50)
	“Status of African swine fever”	PubMed	PS (article)	(51)
	“Eradication of African Swine Fever in Cuba (1971 and 1980)”	By analyzing PS	SS (chapter in a book)	(52)
Dominican Republic and Haiti	“Experiences with Fever in African Swine in Haiti”	By analyzing PS	SS (article)	(53)
	“African swine fever. New developments”	By analyzing PS	SS (article)	(54)
France	“Identification en France· métropolitaine de la peste porcine africaine ou maladie de Montgomery” (article in French)	By analyzing PS	SS (article in Academic University Bulletin)	(55)
	“Peste porcine africaine isolement et identification en France métropolitaine. Données épidémiologiques, cliniques, anatomopathologiques et de laboratoire” (article in French)	By analyzing PS	SS (article in Academic University Bulletin)	(56)
Mainland Italy	“African swine plague. Diagnosis and interventions in the territorial jurisdictions of the Experimental Zooprophylactic Station of Mezzogiorno” (article in Italian)	PubMed	PS (Proceedings of the Conference held in Naples the 1st of March, 1968)	(57)
	“The outbreak of African swine plague in Italy” (article in Italian)	PubMed	PS (article)	(58)
	“African swine plague. Spread, losses and preventive measures in Naples” (article in Italian)	PubMed	PS (Proceedings of the Conference held in Naples the 1st of March, 1968)	(59)
	“Genome Analysis of African Swine Fever Virus Isolated in Italy in 1983”	PubMed	PS (article)	(60)
Malta	“African swine fever in Malta, 1978”	PubMed	PS (article)	(61)
Portugal	“Réapparition de la Peste Porcine Africaine (P.P.A) au Portugal” (article in French)	By analyzing PS	SS (article)	(62)
	Epidemiological research of African swine fever (ASF) in Portugal: the role of vectors and virus reservoirs”	PubMed	PS (Proceedings of the 5th International Symposium on Veterinary Epidemiology and Economics, 1988)	(63)
	“Persistence of African swine fever (ASF) in relation to the economic environment”	PubMed	PS Proceedings of the 5th International Symposium on Veterinary Epidemiology and Economics, 1988	(64)
Spain	“Relationship between the persistence of African swine fever and the distribution of *O. erraticus* in the province of Salamanca, Spain”	PubMed	PS (article)	(65)
	“A case study of an outbreak of African swine fever in Spain”	PubMed	PS (article)	(66)
	“African swine fever eradication: The Spanish model”	By analyzing PS	SS (article)	(67)

The 23 selected papers described historical approaches to ASF eradication and were included in the qualitative analysis (defined as “qualitative synthesis”). Three of these originated from Cuba, 1 from Belgium, 4 from Brazil, 3 from Spain (1 of the three papers retrieved for Spain [ref Table 2, Arias and Sánchez-Vizcaíno (67), was also considered as eligible for Portugal, and was therefore counted once in the methodological approach, but is listed twice in Table 2), 3 from Portugal, 4 from mainland Italy, 1 from Malta, 2 from France, and 2 from the Dominican Republic and Haiti. Table 3 summarizes the literature analysis according to surveillance and intervention strategies.

**Table 3:** African swine fever (ASF) surveillance and intervention strategies for ASF eradication.

**Country**	**YFO/YLO^a^**	**Epidemiological cycle**	**Risk factors for spread or persistence**	**Intervention strategies**	**Surveillance strategies^b^**
Belgium	March 1985/May 1985	Pig to pig	Improper use of infected syringe needle	1. Slaughtering and destruction of animals within the infected farm and culling of infected and not infected traced animals 2. Cleaning and disinfection of farms	1. Syndromic and surveillance on sentinel piglets (AS and PS of pigs at farm) to demonstrate freedom
Brazil	May 1978/Dec 1984	Pig to pig	Contaminated food used to feed pigs	1. Ban of swine movements within and from the affected areas; ban of vehicle and human movements; ban of shows and markets; ban of feeding pigs food waste 2. Inspection at ports, airports, and post offices with more attention to at risk areas 3. Culling and incineration of all swine living in the affected areas 4. Cleaning and disinfecting of vehicles, buildings, and contaminated objects 5. Training campaigns	1. AS at slaughterhouses (serological tests); AS at animal level (special surveillance plan for trade in some at risk regions; test at the origin and destination); AS at herd level (herd certification for trade toward shows and fairs)
Cuba	May 1971/1980	Pig to pig	Contacts between different compartments of pig production characterized by different levels of biosecurity	1971 and 1980 epidemics: 1. Quarantine and movement ban, ban of swill feeding 2. Culling of all infected pigs and in-contact healthy pigs, slaughter of all pigs in neighboring herds (5-km), slaughter of all privately-owned pigs with partial compensation 3. Cleaning and disinfection of buildings, transport vehicles, and personal protective equipment 4. Training in diagnosis 5. Control of entry and departure via railways, roads, ships, and aircraft 1971 epidemic: Radius of 10–15 km around the infected place: 1. Compensation for all culled pigs 2. Transport with high biosecurity measures 3. Movement restrictions of all pigs, commodities, people, and vehicles 4. Complete census of all pigs	1. RBS: division into risk zones based on geographical and political characteristics and density of pork production 2. PS (syndromic surveillance and of pig mortality) 3. AS of pigs at sentinel farms and sentinel abattoirs (specific area) 4. Eradication phase: AS and PS of sentinel pigs at farm level. Test and slaughter approach. 5. Repopulation phase/recovering plan in affected areas: AS on sentinel pigs to demonstrate freedom
Dominican Republic and Haiti	Dominican Republic: 1978/1981 Haiti: 1978/1982	Pig to pig	N.A.	Dominican Republic: 1. Total pig depopulation Haiti: 1. Culling with compensation through Military Army 2. Cleaning and disinfection 3. Training and public education to different stakeholders and cooperation with rural population	Dominican Republic: 1. AS with sentinel pigs for repopulation Haiti: 2. AS with sentinel pigs
France	1st outbreak: 1964/1964 2nd outbreak: 1974	Pig to pig	N.A.	N.A.	1. PS with thermal exploration and blood sampling of positive animals
Mainland Italy	1st epidemic: 1967/June 1967 1969 1983	Pig to pig	Feeding of swine with infected food waste	1. Biosafety and sanitary measures 2. Stamping out in infected farms	N.A.
Malta	March 1978/April 1978	Pig to pig	1. Feeding of swine with infected imported swill 2. Time elapsed between introduction and disease notification	1. Slaughter policy rigorously applied (ban of slaughtering) with compensation; 2. Stamping-out; pig movement restrictions, quarantine of infected and uninfected animals and premises, carcass removal and incineration; 3. Tracing of outbreaks; 4. Prohibition of pork's sale and swill feeding ban	AS at slaughterhouse (serum surveillance) and at farm level.
Portugal	1st epizootic May 1957/June 1958 2nd epizootic April 1960/November 1999	Pig to pig Tick-pig Wild-domestic	1. Transport and improper use of contaminated food waste 2. Uncontrolled movement of animals	1. Stamping-out within infected farms with compensation 2. Cleaning and disinfection of farms, transports, and Personal Protective Equipment 3. Movement restrictions of pigs and pig products from the infected zones or under surveillance; movement ban of pigs and pig products or pig by-products from the infected zone 4. Market and exhibition ban in the infected zones and suspected to be infected; Ban of swill feeding and repopulation	Compulsory notification of suspected and confirmed cases
Spain	1960/September 1994	Pig to pig Tick-pig Wild-domestic	1. Contacts between infected pigs 2. Intimate association between *O. erraticus* and pigs	1. Stamping out in infected farms with compensation 2. Biosafety and sanitary measures: fences, safe disposal of manure, sanitary enclosure 3. Cleaning and disinfection	1. Eradication phase: AS at slaughterhouse and at farm level 2. Repopulation phase: AS in pigs

a *YFO, Year of first occurrence; YLO, Year of last occurrence*.

b *AS, active surveillance; PS, passive surveillance; RBS, risk-based surveillance*.

Each country's eradication history is described below following the chronological order of ASFV appearance.

### ASF Eradication From Portugal

The first outbreak of ASF outside the African continent was notified in Portugal, and probably arose from Angola in May 1957. The spread of ASFV to Portugal was thought to have taken place via contaminated food waste from African airline flights and/or ships docking at seaports, which was fed to pigs (33, 68). This outbreak was effectively controlled and eradicated in June 1958. After 2 years of epidemiological silence, a new outbreak occurred in April 1960 near Lisbon (62), probably caused by the improper use of food waste and waste originating from an infected dead pig whose carcass was not well-buried. From the 1960 epizootic, ASFV spread to many other areas of the Iberian Peninsula (Spain and other areas of Portugal), where it remained endemic for decades until 1994. In 1999, ASF appeared again in the Antalejo region, but it was successfully eradicated. The man-mediated transmission was considered as the most common cause of infection, via the uncontrolled movement of infected animals or the transport of infected animal products from contaminated sites. The uncontrolled movement of animals was probably closely related to the marketing circuits for live animals, as well as the decision-making mechanisms at farm level affecting production and marketing, and which in turn, were affected by the economic environment (64). Furthermore, the complex cycle of the disease, involving probable interaction between wild and domestic suids in the grazing areas (wild boar was considered to represent a potential virus reservoir), and the role of *O. erraticus*, made the eradication very difficult, particularly in outdoor swine production areas where pigsties were used to shelter the free-range pigs (54). In these types of areas, *O. erraticus* was the cause of disease re-emergences, even after disease eradication, as it was the case of the single outbreak in Portugal in 1999 (10). Studies were performed to find *O. erraticus* in the usual resting places of wild pigs; these suggested that the link between soft ticks and wild pigs was not important in the epidemiology of ASF in the wild pig population (69). After tremendous efforts, eradication was finally achieved, jointly with Spain, and specific programs were applied, including the detection of anti-tick antibodies in domestic and wild boars, as well as the destruction or isolation of the pigpens where ticks were present (67).

### ASF Eradication From Spain

The first time ASF was reported in Spain was in 1960 where the disease remained endemic for decades until 1995. The disease spread within the pig sector when the family-type production system was characterized by low-level biosecurity. Extensive husbandry methods used in the management of Iberian pigs made ASF eradication extremely expensive and difficult. In fact, an analysis of the effort to control ASF in Spain in the year 1983 alone estimated costs at 11.4 million Euros (67). After ASF introduction, the pig production system structure was modified to industrial production. Therefore, a specific plan for eradication providing new restrictive policy measures, as compared to the previous plan, was adopted in 1985 (and remained in force until 1995). From 1985 to 1990, the disease was completely confined to southwest Spain. The virus persisted in these areas for several reasons: primarily because of inadequate sanitary and biosafety conditions in outdoor pig production facilities, but also because of the presence of soft ticks (*O. erraticus*), which served as medium and long-term reservoirs of the disease (11), and the presence of an uncontrolled wild boar population, as was the case in Doñana National Park (70). The application of this plan made it possible to divide Spain into an ASF-free region and an ASF-infected region, through a regionalization approach. Afterwards, in 1991, the infected region was divided into a surveillance area (with no acute outbreaks and very few seropositive animals for at least 1 year) and an infected area (66).

During the eradication plan, after outbreak confirmation a protection (with a radius of at least 3 km) and surveillance zone (with a radius of at least 10 km) were established and their radius was adapted according to epidemiological investigations. Movement of live pigs within the two zones was forbidden for 30 days; however, if serological tests proved that the area was negative, movements were allowed within the zones while movements of live pigs outside of the zones were forbidden. All pigs within the protection zone were serologically screened and further screenings were performed in the 3 and 10 km zones, not sooner than 30 days after the preliminary cleaning of the infected holding was completed (67). For holdings that were known to be infested with *O. erraticus*, specific measures were applied, such as no restocking unless special arrangements were made after consultation with the Central Veterinary Administration (67). At the beginning of the program, diagnosis was made through indirect ELISA, which was selected as the best assay for obtaining a rapid and reliable diagnosis (71) for screening, and indirect fluorescent antibody (IFA) assay for confirmation. In the final stages of the program, the National Reference Laboratory developed an improved ELISA containing all the ASFV proteins for better recognizing carrier animals (71). After 35 years of hard work, a key role in disease eradication was played by application of proper biosafety measures, together with a coordinated eradication program conducted with Portugal.

### ASF Eradication From France

In April 1964, the disease appeared in France, with the notification of five outbreaks: one in the southwest, three in the southeast area bordering Spain, and one in the Bretagne region. The disease entered France through the illegal introduction of infected pigs from Spain (55), but it was eradicated in May 1964 (56). No surveillance and control measures were described in literature. A second outbreak was notified in 1967, and a last outbreak in 1974 in the southwestern part of France, in the Atlantic Pyrenees region (56). In this last case, the movement of infected animals traded from Spain probably caused the outbreak. Classical surveillance on clinical suspects was applied together with thermal exploration, followed by blood sampling in case of positivity (56). The outbreaks observed in France were characterized by low virulence both from an epidemiological and a clinical point of view (55, 56).

### ASF Eradication From Mainland Italy

In Italy, an extensive outbreak was recorded in Rome, in the Lazio region (58), during the first month of 1967. The disease appeared because of the practice of animal feeding of raw urban food waste (58). This first epizootic affected 28 provinces with 205 outbreaks and was contained through the culling of 99,458 pigs. This intervention of the veterinary services was severe and immediate, so that the wild boar population located in the area surrounding the outbreaks remained free from the infection (58). After the first outbreak confirmation in 1967, an infected zone (Municipality of Rome) (58) and a protection zone (the entire province of Rome) were established (57, 58). A strong collaboration was set up among different Italian ministries, the national authorities, the OIE, and the veterinary services.

Afterwards, the disease spread to Naples through illegal commerce of infected pigs and swill feeding (57). Italy experienced a recurrence in 1969 and then in 1983, when ASF was lastly reported on a farm near Turin (57, 59). All these outbreaks were controlled by a rapid slaughter policy and each time the disease was eradicated. The disease was swiftly controlled and eradicated from mainland Italy through the interdiction of swill feeding and the massive stamping out of all infected holdings (57, 59), with compensation (59) and proper cleaning and disinfection measures. Repopulation was done after 6 months from the date of the culling of the last animal. During the post-eradication phase, no particular surveillance measures were described in literature.

The situation in Sardinia is not described here, because eradication has not yet been achieved. Since 1978, this Italian island has been the only European ASF-infected area (14).

### ASF Eradication From Cuba

The disease was never been diagnosed in Central America until 1971 when the virus was introduced to Cuba and then spread within the country through privately-own pigs, private vehicles and transport, or by swill-feeding (50). Although firstly reported in May 1971, the authorities admitted its presence only in late June 1971. The length of time that elapsed between the actual occurrence and the notification was due to the time required for diagnostic support provided by Russia and Canada (51). The first epizootic occurred in a fattening holding in the province of Havana, which received animals mostly from the State's swine units (specialized porcine farms) and from some privately-owned pigs (farms in which the number of pigs per unit is limited and pigs are only for personal consumption). The late diagnosis allowed ASF to spread throughout the whole province of Havana and was confined to the province (51). The success of disease confinement was likely attributable to the involvement of several technical working groups (National Institute of Agrarian Reform, Ministry of Public Health with different Epidemiology groups, the Ministry of Home Affairs, the Ministry of Industrial Feeding, and the University of Havana) with different skills, and clear and defined tasks in the command chain (51). Furthermore, the Cuban authorities set up a dedicated Control Commission with national and international bodies (51).

On 26 January 1980, a second epidemic occurred in the eastern region of the island, in the municipality of Barcoa, in proximity to the Republic of Haiti (52). Initial analysis indicated that the disease entered Cuba by means of food products brought by Haitian immigrants arriving in an uncontrolled immigration (52). The overall loss was estimated to be 9,359,414 US Dollars. Surveillance on sentinel pigs to prove freedom from ASF started at the end of September 1980 (52).

Various control measures were applied for eradication both in the first and in the second epidemic. In infected premises, several measures were applied: strict quarantine, culling of all sick pigs and in-contact healthy pigs, or pigs suspected to be infected; disinfection of both infected premises and the area surrounding the outbreak; killing of rats, dogs, cats, and other animals that could have been mechanical vectors of the virus; treatment of the herbage and the soil with calcium hypochlorite; wood burning in buildings that could not be properly disinfected, and finally repopulation activities (51). Around the infected premises, in an area with a radius of 10–15 km, compensation was provided for all culled pigs, and special transport, with high biosecurity measures, was arranged for these pigs to official slaughterhouses; all the equipment used in the pig units were cleaned and disinfected. Moreover, movement restrictions of all the pigs and related commodities, both in the private and in the state sectors, people, and vehicles entering swine establishments, in addition to a complete census of all pigs in Cuba, were enforced (52).

### ASF Eradication From the Dominican Republic and Haiti

In the Dominican Republic, ASF entered in February 1978, and subsequently it entered Haiti in December 1978, with the classical form characterized by high mortality. The disease probably entered the Dominican Republic through infected pork scraps from an international flight from Spain and spread rapidly throughout the country (54). When the disease was confirmed in the Dominican Republic in July 1978, an agreement was reached between the two countries to slaughter all swine within 15 km on both sides of the border (53). With the cooperation of the Food and Agriculture Organization (FAO), the United States, and the International Development Agency, all outbreaks were eradicated from the Dominican Republic and total depopulation was achieved. In July 1980, in an effort to detect the residual virus, sentinel pigs were introduced for repopulation Up until September 1981, no cases of clinical disease were recorded, and all serological tests of newly introduced pigs were negative (54).

While the Dominican Republic endorsed an eradication program, Haiti took no actions at the beginning of the outbreak, either because of lack of funds or appropriate animal health infrastructure. With the support of four countries, the U.S. Animal Health Association, the U.S. National Pork Producers Council, and the National Association of State Department of Agriculture, an eradication program was drafted and started in Haiti in April 1981. It comprised 4 phases: (I) Six months of planning and information/public education; (II) Slaughter/compensation; (III) Cleaning and disinfection and raking; and (IV) The establishment of pig sentinels. Eradication was possible through the elimination of the swine population with the support of the Haitian Army, but the public information program was considered crucial for gaining the cooperation of the rural population. Haiti declared eradication on 28 April 1982 (53). Furthermore, the Pan American Health Organization (PAHO) and FAO defined emergency measures and training activities for field and laboratory, for the early identification of cases, and a specific program was established to coordinate ASF control for Latin America and the Caribbean. Together with the government of Jamaica, PAHO worked very closely with the veterinary services of Haiti to strengthen their capacities, quarantine measures, to review regulations governing entry of pigs and pork products into the country, to provide training involving customs, police, and animal health personnel, and to investigate deaths in pigs (53).

### ASF Eradication From Malta

ASF was first notified in Malta in March 1978, after an outbreak involving infected imported waste illegally fed to animals. The first cases were notified in pigs in fattening premises, which had bought weaners from swill-fed premises where the disease was well-established, indicating that it had probably been in Malta for at least a month before diagnosis and notification. Therefore, ASF rapidly spread throughout the country affecting 25,100 pigs and 304 premises. In addition to the spread of virus in contaminated swill the movement of weaners from infected swill feeders was a key means of spreading the infection. In the early stages, farmers voluntarily depopulated their premises. A serum survey was carried out at slaughterhouse and at farm levels. By August, the pig population was reduced to one-third. A rigorous policy of slaughtering with compensation was applied in the island leading to the disease confinement and finally eradication. This result was achievable thanks to the restriction of pig movements and the elimination of the large number of infected pigs once the slaughter policy was adopted (61). After 10 months from the notification, at the end of January 1979, there were no pigs left in Malta (61).

### ASF Eradication From Brazil

First notified in Río de Janeiro, in the municipality of Paracambi, in May 1978 (46, 49), Brazil experienced ASF due to tourism between Spain, Portugal, and Brazil, and the illegal trade in leftover food from flights landed in Río de Janeiro that was used for swine feeding (46, 49). Brazilian authorities declared an animal health emergency even before the laboratory results became available (49) and rapidly applied proper control measures. The disease spread due to contaminated food used to feed pigs housed on farms with low-level biosecurity (thus, the epidemiological determinant was a social factor), and through contaminated classical swine fever (CSF) vaccines that arrived in Paraná via the municipalities of Ourinhos and Jacarezinho in Sâo Paulo State (46, 49). During the emergency period (1978–1979), a federal level working group and an official laboratory for ASF diagnosis (ASFDL) were set up. The ASFDL was a paramount tool for the adoption of best eradication practices, providing information on ASFV heterogeneity (low- and high-virulence strains) (48). During the emergency period, all the actions were integrated between the Ministry of Agriculture, the Ministry of the Army, and the Military police. Several actions to control the disease were implemented, such as the immediate notification of cases to neighboring countries with which Brazil had bilateral agreements, and to the OIE and the FAO. Other measures applied included the destruction of clandestine deposits of food waste in the cities, with the removal and destruction of all food waste, the ban on sale of animals and pork products and on feeding of food waste; control of pig movements, with a ban on exhibitions, fairs, and other events of aggregation; the setting up of check-posts; census activities in the focal area; culling and immediate cremation of pigs within the affected areas; repopulation 6 months after the last eliminated case, and at least two rounds of disinfection of the affected premises, with the reintroduction of sentinel pigs free from ASF and vaccinated against CSF; active training and social programs related to preventive measures (farmers and veterinarians received phone numbers for free direct calling, so that they could notify the authorities as easily as possible).

In November 1980, a vast national program was launched which aimed at eradicating ASF and controlling CSF simultaneously in a joint effort. The program's activities had characteristics in common with the previous phase, with exception of vaccination against CSF (48). The technical and financial support for the program (from 1980 to 1987) and the establishment of diagnostic facilities for ASF surveillance were only possible jointly with the Federal University of Río de Janeiro, the financial support by the FAO and OIE and the Ministry of Agriculture (46, 49). The program was applied throughout the country, with selection of the Southern region as a priority area, due to its pig density. The program consisted of three stages of actions, namely, attack, consolidation, and maintenance stages.

The attack stage, applied between 1980 and 1984, consisted of targeted surveillance at ports, airports (mainly for flights coming from at-risk areas), control of internal movements, inspection comprising serological tests both at the place of origin and the destination, in addition to active surveillance both in pigs for slaughter at the slaughterhouses and in breeding centers associated with certification of the sanitary status of farms as ASF-free. Other actions, such as systematic vaccination against CSF, the restructuring of regional laboratories, training and awareness in animal health, and the implementation of a national information system, were also adopted.

The consolidation stage, which was in force between 1984 and 1986, aimed to identify new possible outbreaks through maintenance of the surveillance system and control of animal movements. The last stage, the maintenance stage, began in 1987 by way of the application of the general surveillance system set up for pig diseases (46, 47).

An activity named “garbage operation” within the eradication campaign was noteworthy; this was based on the registration and elimination of pigs kept in public garbage plants and slums performed with the help of the Ministry of Health and the Military Police (46–48). This action was responsible for the end of the transmission cycle of the disease within non-industrialized breeding programs (46, 47). Between November 1981 and September 1984, no new outbreaks were reported, and Brazil regained its status of ASF-freedom in December 1984. The prompt identification of the disease, the rapid notification, the swift implementation of actions, the social communication with farmers, the active participation of breeder associations in the democratic decision process, the government support (49), the financial compensation, the collaboration with international organizations (FAO and OIE), the stamping-out policy within the infected and suspected areas, the self-limiting nature of the disease in low-density pig farms, and the absence of soft ticks (Brazil has the advantage of an absence of complicating factors, such as wild hosts and vectors) (46, 49), led to successful eradication of the disease within 6 years (46, 47).

### ASF Eradication From Belgium

The first case of the ASF in Belgium was reported in West Flanders in March 1985. The virus was probably introduced through infected pork from Spain that was fed to a wild boar. Afterwards the spread occurred through direct contact (trade) of infected animals and improper use of infected syringe needles (45). The disease was eradicated in all 12 infected farms within the country during 3 months after its first detection. The slow spread of the virus (due to epidemiological circumstances) together with the severe control measures applied led to eradication, which was declared in September 1985. The absence of viral circulation was confirmed by a large serological survey after the last confirmed case. The eradication goal was achieved by combining severe control measures with active and passive surveillance at farm level. Serological surveillance, aimed at eradicating the disease, was applied to both infected and not infected herds, and to several farms with indirect contact with those suspected to be infected. The interval between disease confirmation and eradication dates was short: for 5 of the 12 infected farms, the date of confirmation and the eradication date coincided, while, in other cases, a maximum of 5 days elapsed between confirmation and eradication. In the literature, no specific risk factors for maintenance were described given the fast eradication achievement (45).

### ASF Eradication From the Czech Republic

The first ASF positive carcass was found in Príluky, Zlín district, in an inhabited area of the Czech Republic, in June 2017. This epidemic focal incursion of ASFV involved a limited wild boar population and progressed slowly in space. Since its first introduction until December 2017, the disease spread slowly at a rate of 0.5 km/month, despite the high wild boar density (8–10/km^2^) (72). The infected area was located 30 km from the Slovak border and 80 km from both the Austrian and the Polish borders. From 2014 to March 2019, 4,296 wild boars found dead were tested for ASF, of which 211 tested positive. The last ASF-polymerase chain reaction-positive case in hunted wild boar was found in February 2018, and the last two positive cases in carcasses probably dead 4–5 months before discovery were identified in April 2018 (72).

Nationwide passive surveillance started in 2014 and was applied to all dead pigs found throughout the country. It proved to be a key factor in early detection of ASF that enabled an immediate and effective response (72). The strategy for successful eradication was based on the definition of different wild boar management zones according to a certain level of risk into three areas:

1. An infected area divided into (1a) zone with low risk and inside it a (1b) zone with high risk defined by a polygon of 159 km^2^ estimated on wild boars' year-long home range. In addition, fences were built within the high-risk zone to delimit an area of 57 km^2^ where the total depopulation with high biosecurity measures was performed by policy snipers specially trained for hunting in biosecurity;

2. An intensive hunting area of 8,500 km^2^, excluding the Zlín district (72), on the outskirt of the low risk zone;

3. and the rest of the country.

After first confirmation of ASF in June 2017, hunting was regulated firstly through a ban within the infected area, then it was allowed only in infected area of the low risk zone, then it shifted from the trapping of wild boar in the high risk zone to individual hunting in the same zone in the infected area (73). The measures and approaches used after the outbreak's confirmation differed depending on the risk of infection. The success of ASF eradication in the Czech Republic relied on the management zones' demarcation, enhanced passive surveillance of dead wild boars through intensive and systematic searching and removal of carcasses, a ban on driven hunting, motivation for hunters through financial rewards and compensation, high biosecurity during hunting and sampling collection in the infected area, disposal of hunted wild boars from the infected area to/selected//definite rendering plants, effective hunting in the infected area by snipers, and awareness training campaigns and education of hunters, veterinary services, and the public (72).

## Discussion

The history of ASF is close to be one century long and in this period it was possible to collect several key elements from an epidemiological point of view. The disease was confined to Africa until the end of the 1950's when it appeared in Portugal in 1957. After 2 years' silence, the disease appeared again in Lisbon in 1960 and spread to the Iberian Peninsula and to other European countries: Spain in 1960; France in 1964, 1968 (74), and 1974; mainland Italy in 1967, with recurrences in 1969 and 1983; Malta in 1978; Belgium in 1985; and the Netherlands in 1986 (75). Between 1971 and 1980, ASF appeared in several American countries: Cuba, in 1971 and again in 1980; Brazil in 1978; the Dominican Republic in 1978 and Haiti in 1979 (67, 76). In the past, in both European and American countries the disease has been successfully eradicated, whereas in the current epidemics, only the Czech Republic managed to eradicate the disease in wild boar population (72).

Eradication was possible in different epidemiological contexts, with intensive or extensive swine breeding, and also in areas with the presence of or with an intimate association between *O. erraticus* and pigs, such as in Portugal and Spain (77). Nevertheless, it should be considered that eradication of *O. erraticus* ticks is extremely difficult (78) and epidemiological studies carried out in infected areas of Spain highlighted that, once ASF was eradicated from the domestic pig population, it also disappeared from the wild pig population. Therefore, most probably, the role of the wild boar population was not relevant in the spread of the disease (65) or in the persistence of viral circulation. Based on epidemiological data from the Spanish scenario, the role of carriers in virus dissemination seemed to be not so important when appropriate control measures were put in place (66).

Eradication was sometimes difficult, long-lasting, and costly, as demonstrated in Spain, where the disease was present for 35 years before its eradication (9) or in Portugal, where ASF was also present for decades. It was reached in a reasonable, or very short, period in Cuba, Brazil, Belgium, Malta, mainland Italy, France, the Dominican Republic, and Haiti due to the application of classical preventive and surveillance measures. Cases of particular interest were represented by France and Belgium. In France, the eradication was possible through the application of classical measures, but was facilitated by the presence of the Pyrenees (68), which acted as a natural barrier and minimized ASFV spread, leading to the occurrence of local epidemics (45) that were promptly eradicated. In Belgium, both the favorable epidemiological circumstances leading to slow viral spread and the short interval between the disease confirmation and eradication in most of the affected farms, enabled disease eradication in 6 months.

The recent experience of the Czech Republic was noteworthy, because it is the sole country officially declared free from ASF in recent years. Early detection and strict new measures in wild boar populations have been applied to prevent ASF spread, and containment efforts have recently met with success using different wild boar management zones; leaving wild boar in the infected area and by removing the carcasses, and depopulating around the infected zone (i.e., the fenced area, high- and low-risk areas, and intensive hunting area) (72). When the infection levels estimated from the carcasses decreased, depopulation was also put in place in the infected area. As a matter of fact, 10 months after discovery of the index case, ASF had been confined to a very small territory in the Czech Republic and has apparently not spread. Although eradication has not been achieved in the other involved EU countries, the Czech Republic experience can be considered to be a first successful attempt in disease control in an epidemiological scenario characterized by a small cluster of infections in wild boar population. As in the past, classical surveillance strategies and control measures continue to be valid tools for disease control and eradication. Also the experience of Belgium deserves special mention. In this country the disease was absent since 1985 but reappeared in a confined area on 13 September 2018 in wild boars, likely due to human activities (79). Even though Belgium has not yet been completely declared free from the disease, the control strategy applied was proving effective in limiting ASFV inside the affected area and confined to the wild population. This was possible thanks to preventive culling of all domestic pigs and captive wild pigs in the provisional “infected zone” extending over 630 km^2^ along with a ban on the repopulation. In the rest of the country enhanced passive surveillance in all pig holdings, training of veterinarians, increased biosecurity measures and prohibition of assembly of pigs were assured. After the replacement of the provisional “infected zone” with zone II and I according to the Directive 2002/60/EC, specific additional and more stringent measures than those imposed by EU were applied within the three operational zones (an infected area bounded by two concentric peripheral zones called “reinforced observation area” and “vigilance area”). The ban of hunting and wild boars' feeding, the active and systematic searches for dead wild boar with immediate carcass removal and transport to the principal collection center then to the rendering plant jointly with soil disinfection were applied. Furthermore, a network of concentric fences was built with the dual purpose of slowing down the spatial diffusion of the disease and defining corridors aimed at collecting wild boars to be depopulated by avoiding their dispersal. The depopulation was carried in all the three zones by hunters who had received specific training on biosecurity procedures and compensation.

These results are sustained by a recent review (44), in which different surveillance and intervention strategies for ASF and their effectiveness were assessed, based on expert opinion. The authors identified surveillance and intervention strategies perceived as being the most effective. Among the 20 surveillance strategies identified, passive surveillance of wild boar and syndromic surveillance of pig mortality were considered to be the most effective for controlling ASFV spread, whereas culling of all infected herds and movement bans for neighboring herds were considered as the most effective intervention strategies. Regarding wild boar populations, active surveillance, and carcass removal were rated as the most effective surveillance and intervention strategies, respectively, but they were also considered the least practical, suggesting that more research is needed to develop more effective methods (44).

Currently, ASF is still present in some geographical areas of eastern and northern Europe and it is endemic in Sardinia (Italy) (76). In contrast to countries that achieved eradication, the Italian island of Sardinia is the only European ASF-infected area where the disease has been endemic since 1978 (14) as a consequence of the first European epidemic wave. In the past, the arduous habitat and the old practice of “brado” (free-range pig keeping, illegally maintained in public concession areas in traditional breeding practices) (40) on state-owned pastures represent an essential epidemiological link between the domestic pig and the wild boar population in the central-eastern part of the island (14). The overlap of these epidemiological conditions, together with other social and economic factors, represent the main obstacle to eradication. Recently, the fight against illegal breeding was intensified by mandatory culling and economic support to improve the farms' biosecurity levels, aiming to promote high quality pig products in compliance with local traditions (40). At present, the levels of infection in the population of feral pigs are decreasing and wild boars are considered a source of infection that is of secondary relevance to the presence of illegal wild pig breeding. Therefore, a hunting regulation plan, aimed at increasing the biosecurity level of hunting, as well as effective monitoring of the epidemiological situation were applied, and additional actions to limit wild boar population density were promoted.

Furthermore, the significant improvement of the epidemiological situation in domestic pigs in Sardinia (no disease outbreaks were registered from the beginning of 2018 until June 2019) was mainly attributable to improved control of illegal free-ranging pigs and better biosecurity on pig holdings (80, 81). On the whole, the significant progress in ASF control currently recordable (80, 81) demonstrated that it is not possible to control the spread of the infection underestimating the rules yet expressed in the EU legislation. A strict biosecurity approach on pig holdings, an effective animal registration as well as the contrast of illegal practices are all burdensome measures difficult to implement, but definitely essential. Actually, the application of this strategy includes a paradigm shift in traditional practices and in human behavior that are possible only by a great effort in informative campaigns.

It is noteworthy that only in one occasion ASF has spread outside Sardinia: in Piedmont, in March 1983 (60), affecting only three farms. This was due to wild boar meat imported from Sardinia. Strict quarantine and slaughter measures limited the spread of the disease in Piedmont and the outbreak was successfully eradicated (60). Therefore, the presence of ASFV in the island seems to pose a limited risk to the pig sector of ASF-free European countries (82, 83).

Similarly, as in Sardinia, humans' role was also considered to be relevant in the disease spread in the Northern European scenario (Table 1). Epidemiological analysis of ASF in the Baltic States and Poland, performed by the European Food Safety Authority (EFSA), aiming at estimating the relationship between the presence of ASFV in the wild boar population and environmental/biological factors, indicated that the human-mediated spread of ASFV played a critical role in the epidemiology of the disease. It was concluded that reduction of the wild boar population and carcass removal to stop the spread of ASFV in the wild boar population were more effective when applied preventively. The pressure exerted by outbreaks both in the domestic and in the wild population in the former Soviet Republics eventually involved European Union Member Countries, such as Poland and the Baltic Republics (Estonia, Lithuania, and Latvia) that were progressively affected from the beginning of 2014 to date (26). The analysis of available data regarding the incidence of ASF outbreaks in certain non-EU Countries authorizes the suspect of lack of information. In this context, it is quite impossible to properly investigate the relevance of multiple introduction of the virus in the epidemiology of this disease. However, ASFV does not recognize country borders and if considering the viral circulation in connected wild boar populations, progress of the virus in the border areas can be foreseen. On the other hand, it is pleonastic to remark that in the case of single introduction of the virus in a previously free territory or, better, in the case of focal spread in a very limited area, the chances to promptly reach the disease eradication are significant, especially if associated with an early detection and an efficient application of restriction measures.

Unlike the Eastern Europe scenario (Table 1), where the backyard network of farms with low-level of biosecurity was the main reason for the local ASF transmission, and the transfer of food products was the probable cause of long-distance infection (84), in the Northern Europe scenario, the wild boar population played the main epidemiological role (11, 85). The main risk factor facilitating the persistence of infection in Northern Europe was the contamination of the forest areas where the infected carcasses of dead wild boars lay for many months (23).

Results of this review also confirmed that the role of wild boar was generally supported by other factors (the presence of tick vectors in Portugal, human-mediated in the Baltic states, human factors in Sardinia, etc.). However, the density and population dynamics of wild boars currently represent a new challenge to solve. A scientific opinion was recently published by the EFSA (86), with the aim of providing an estimate of the wild boar densities in the EU, identifying thresholds in the wild boar density that do not allow sustaining the disease in different settings, and reviewing wild boar depopulation methods or population density reduction methods. They reported that passive surveillance on dead wild boars is the most effective and efficient method for early detection of ASF in free areas. Preventive measures for reducing and stabilizing wild boar density, before ASF introduction, will be beneficial both in reducing the probability of exposure of the population to ASFV, and the efforts needed for potential emergency actions (i.e., less carcass removal) if an ASF incursion were to occur.

History of ASF eradication indicates that this infection may appear in different ways, although the ASFV can shows very limited genetic diversity (87). In fact, in continents where only genotypes I and II have been circulating the genetic diversity among isolates collected over long time periods and from different geographical regions was very limited (87), in contrast to isolates from the sylvatic cycle in East and South Africa characterized by greater genetic diversity (34, 87, 88). Furthermore, large differences highlighted in the virus genome (89) do not seem even to influence the ASF epidemiology in terms of mortality, morbidity, and resistance; if ever, the interaction with the hosts and the environment are more affecting the virulence expression: in fact, recent studies (89) indicate that the virulence may be modified as a consequence of the extended exposure of the host population to the infection.

As a matter of fact, ASF can occur as an epidemic, making long jumps, crossing borders, and even passing through continents; very often the first occurrence of the disease is a harbinger of rapid dissemination in naïve populations, whereas, in the past, certain outbreaks were immediately resolved by applying restrictive measures to the infected farms due to early detection. On the other hand, the viral spread could evolve in an endemic manner, in both the domestic and wild populations, due to its persistence in vectors or wild hosts, or due to human factors. In these cases, the eradication strategies are less effective and very expensive to apply in terms of direct and indirect costs. These lessons have been widely underestimated; nevertheless, we are learning that new sources of infection, which can create new scenarios, should be considered in risk analysis: the most important factor, which has been underestimated in the past, is the human factor. Probably, when early detection is applied along with strong awareness campaigns, this factor could have a limited effect. Nowadays, globalization, the movements of people, trades, and other similar factors, are currently contributing to increase the risk of ASF spread. Therefore, the most relevant lesson that should be considered is that the human role, human behaviors, social, cultural, and historical factors involved particularly in endemic areas, are crucial in any step of ASF control. Besides the wild boar population and habitat, the current European epidemiological situation also implicates humans as the main cause of both long-distance transmission and virus introduction to domestic pig farms (90). Therefore, in addition to biological aspects, it becomes crucial to include social science when planning prevention, control, or eradication measures (90). The countries that succeeded in eradicating the disease teach us that prompt eradication can be achieved only by applying early detection and proper control and intervention strategies, as foreseen by the EU legal framework for ASF. In fact, the prompt identification of cases allowed rapid eradication of the disease in the case of mainland Italy, Malta, and Belgium, and the epidemiology and laboratory networks played an important role in gathering data and providing epidemiological interpretation. Where a well-structured collaboration among different institutions of affected countries was put in place in the cases of Brazil, Cuba, the Dominican Republic, and Haiti, mainland Italy, Portugal, and Spain, successful eradication was achieved even in scarce economic contexts. Effective eradication was achieved when task forces of experts and appropriate communication skills, appropriate to that historical period, were applied. Instead, drastic measures applied for eradication of ASF in Cuba, such as killing of rats, elimination of dogs, cats, and other animals that could have acted as mechanical vectors of the virus, would be inapplicable in EU countries.

A final consideration of topical interest involves data collection on ASF at the European level. Linking outbreak information with surveillance and laboratory data, with the pathogen characteristics, would help in understanding the disease and its genetic dynamics in the spatial and temporal context and allow improvement of control and eradication strategies. At present, these data, if available, are usually collected at country level, with several information systems in place even in different regions of the same country, having different aims, and owned by different organizations. At EU level, data on the outbreaks of notifiable animal disease are currently registered into the Animal Diseases Notification System (ADNS) (91). However, the quality of data concerning each outbreak is currently poor, especially for data indispensable for evaluating the progression of the disease. Moreover, the information is often not linked to surveillance and laboratory data. The collection of data and information on ASF surveillance is fragmented even within a given country; this does not support the progression of control and eradication of the disease. Moreover, while data on farmed susceptible species and information on herds, densities, and locations (geographical coordinates) are stored in well-structured databases and information systems (92), densities and geographical distribution of wild susceptible animals are collected by the EU countries with different systems, each having their own specific characteristics with respect to the methodology used, the type of data acquired, the repository implemented, and data accessibility. This is of particular concern given the spread of ASF from Eastern Europe areas. In this framework, the ENETWILD EFSA funded project is attempting to develop standards for data collection, validation, and to create a data repository (81). Moreover, starting from 2019, EFSA has conducted a project with the support of volunteers EU Member States, aimed at building a harmonized and coherent platform for exchange of surveillance and laboratory information on ASF, lumpy skin disease, and Avian influenza (93). A coherent and harmonized data collection system would allow EFSA to perform proper risk analysis, with the aim of improving surveillance systems, and achieving eradication of the diseases.

## Conclusions

We found documented reports for nine countries all over the world (Africa excluded) that had to manage ASF, as a whole, between 1954 and 1999 and they were able to reach the eradication. The eradication was achieved in few months or in more than 35 years.

The ASF infection demonstrated, over the years, to be really difficult to be eradicated. The sole continuous presence of viral circulation in Africa gives the evidence that the risk of new incursions of the disease are possible and the current epidemiological situation multiplies the chances of ASF virus spread all over the world.

The first epidemic wave started in the 50s', as such as the recent experiences of Czech Republic and Belgium, lead us to be optimistic: the virus first incursion is generally referable to an epidemic form that, in case of prompt and rigorous containment, can be kept under control or eradicate in a reasonable period of time.

Conversely, the disease, if not properly controlled, can easily turn into the endemization, as confirmed after the second epidemic wave began in the Caucasus region in the 2007, when the disease became endemic, involving also other countries.

African swine fever can be controlled and eradicated through classical surveillance and control measures, as demonstrated in the past experiences of countries worldwide if the main epidemiological target remains the domestic pig population. Classical measures are based on disease control methods, including surveillance strategies, epidemiological investigation, tracing and culling of pigs in infected holdings, in combination with strict quarantine and biosecurity measures on domestic pigs, holdings, and the control of animal movement. These measures are currently in force within the EU legal framework for ASF control, as laid down by Council Directive 2002/60/EC (94). The Directive also requires that Member States develop and implement plans for the eradication of the disease (95). These measures were effective in addressing a number of outbreaks, as exemplified in the Czech Republic's first experience of ASF. However, evidence also suggest that this strategy is difficult to sustain for a long period in endemic situations, such as in the Baltic States and Poland, where the disease affects larger areas. A successful strategy in this scenario has not yet been found.

In fact, the experiences collected in recent years demonstrated that the involvement of wild boar population in the viral spread hampers the eradication and, for sure, it is a relevant risk factor facilitating the virus spread across the country borders.

Therefore, an efficient strategy for ASF prevention or control should be based on deep knowledge of target domestic and wild population, of environmental conditions and type of swine sector. Nevertheless, all the strategies have to take in count that the disease knows no bounds and a common policy should be defined.

Finally, unlike in the past, considering the increase in globalization of animals and food products trade as well as of human beings, the effective collaboration among EU and non-EU neighboring countries would allow the definition of standards for data collection and validation, preventing new virus incursion.

## Author Contributions

All authors contributed to the manuscript. FF helped to conceptualize the study. MD and FF designed the study and defined the research objectives. MM and MD defined the research methodology. MD and FF retrieved the papers, compiled all information, and wrote the manuscript. MD, FF, MM, PT, and SI contributed to writing the manuscript. PC and PT contributed to final revisions. All authors contributed to critical review of the manuscript and approved the final version.

## Conflict of Interest

The authors declare that the research was conducted in the absence of any commercial or financial relationships that could be construed as a potential conflict of interest.
